# Turbo-Satori: a neurofeedback and brain–computer interface toolbox for real-time functional near-infrared spectroscopy

**DOI:** 10.1117/1.NPh.4.4.041504

**Published:** 2017-10-06

**Authors:** Michael Lührs, Rainer Goebel

**Affiliations:** a Maastricht University, Faculty of Psychology and Neuroscience, Department of Cognitive Neuroscience, Maastricht, Netherlands; b Brain Innovation B.V., Maastricht, Netherlands

**Keywords:** functional near-infrared spectroscopy, real-time, neurofeedback, brain–computer interfaces, support vector machines, general linear model

## Abstract

Turbo-Satori is a neurofeedback and brain–computer interface (BCI) toolbox for real-time functional near-infrared spectroscopy (fNIRS). It incorporates multiple pipelines from real-time preprocessing and analysis to neurofeedback and BCI applications. The toolbox is designed with a focus in usability, enabling a fast setup and execution of real-time experiments. Turbo-Satori uses an incremental recursive least-squares procedure for real-time general linear model calculation and support vector machine classifiers for advanced BCI applications. It communicates directly with common NIRx fNIRS hardware and was tested extensively ensuring that the calculations can be performed in real time without a significant change in calculation times for all sampling intervals during ongoing experiments of up to 6 h of recording. Enabling immediate access to advanced processing features also allows the use of this toolbox for students and nonexperts in the field of fNIRS data acquisition and processing. Flexible network interfaces allow third party stimulus applications to access the processed data and calculated statistics in real time so that this information can be easily incorporated in neurofeedback or BCI presentations.

## 1 Introduction

In recent years, the developments of brain–computer interfaces (BCIs) based on functional near-infrared spectroscopy (fNIRS) have been continuously evolved establishing its use for human–computer interaction (HCI)^1^^–^^3^ as well as for brain state decoding^4^^–^^8^ and neurorehabilitation.^9^ fNIRS is a very attractive method for these applications, because it is a noninvasive, portable, and low-cost technique, compared to methods like functional magnetic resonance imaging (fMRI), while also relying on the blood-oxygen-level-dependent (BOLD) effect.^10^ The increased usage of fNIRS for HCI and BCI applications is only possible because previous research improved the signal quality and reliability of fNIRS measurements.^11^ A lot of the BCI research in this field was based on individualized analyses using various machine learning methods such as support vector machines (SVMs) or other classifiers.^12^ While these methods allow the use of BCIs, they usually do not providing insights in the signal quality and trial-by-trial performance of the participant. We developed a software package called Turbo-Satori bridging the gap between comprehensive analysis methods and the detailed inspection of fNIRS signals in real time during an ongoing experiment. This possibility is very important since it allows real-time quality assurance by inspecting individual fNIRS channels and the overall signal quality of all channels at the same time. Especially when using neurofeedback routines, the quality of individual channels is crucial because they can be used as the basis for the neurofeedback signal, translated for example to the level of a thermometer display.^13^^,^^14^ But also in other BCI applications, transparent information and statistical data about the signal quality of individual channels are important to relate it to the overall performance. Therefore, the mentioned features allow several potential use cases for Turbo-Satori requiring minimal implementation time because of the available interfaces and clearly structured user interface. We provide different use case examples for Turbo-Satori including SVM-based left–right finger tapping discrimination, neurofeedback example experiments, and BCI application examples. Another aspect is the usability of the software which needs to fulfill the requirement of integrating and controlling complex routines while assuring its ease of use. Such a system allows even early stage researches to perform advanced real-time fNIRS-based BCI experiments with relatively little effort. In this paper, we will cover all major aspects of the software starting with the unique user interface and controls followed by specific preprocessing and analysis routines.

## 2 Methods

Turbo-Satori is written in C++ and uses different libraries and frameworks such as Qt^15^ and Eigen^16^ as well as a collection of useful C++ classes for digital signal processing.^17^ While developed for cross-platform use, the software currently runs only on the Windows platform in combination with devices from the company NIRx (NIRx Medical Technologies, LLC, 5670 Wilshire Blvd., Suite 1800, Los Angeles, California). The next section describes the user interface of Turbo-Satori that is designed to focus mainly on rich time course information and ease of use, especially for research investigating individual fNIRS channels.

### 2.1 User Interface

**Fig. 1:**
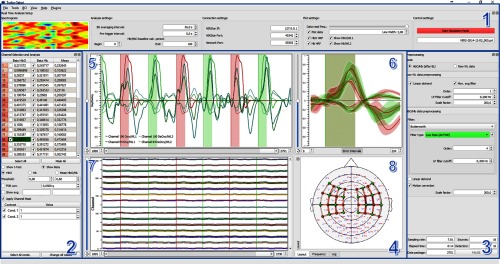
The graphical user interface of Turbo-Satori. The interface consists of four parts: 1. The real-time analysis setup window, which includes configurations to set up the real-time analysis as well as connection, plotting, and control settings. 2. The channel selection and analysis window. It contains the basic parameters and table to perform real-time analysis of the available channels. 3. The preprocessing window allows to change the preprocessing parameters for raw wavelength data as well as for the HbO/Hb converted data. Additionally, the status information is shown in the bottom of the window. 4. The main application window provides three different plots and a layout view, frequency spectrogram and log window area (numbers 5 to 8) showing useful information or the layout of the sources, detectors, and channels. 5. The time course of the selected channels (or average) for raw wavelength 1 and/or 2, HbO and/or Hb signal. 6. The event related average of the selected channels time course. 7. The combined plot for all marked channels. 8 The layout view, (layout shown in this figure, see tab bar at bottom).

The user interface of Turbo-Satori mainly consists of four parts with specific settings and options. These interface parts can be freely rearranged or hidden to be able to limit the data representation to only the currently desired information. A screenshot of the running application is shown in Fig. 1. The first part of the interface (see number 1 in Fig. 1) allows the user to configure global settings of analysis, controls, and plotting. It also contains the control settings to start/stop the real time, online, or offline analysis. On the left side of the interface, all available channels are displaying the beta value of each channel at the current point in time using the currently defined contrasts. The arrangement of the channels starts with first source and first detector in the top left corner to source and detector in the bottom right like the definition in the NIRStar data acquisition software allowing the user to immediately observe whether the expected pattern is present ensuring data quality.

The second part of the interface covers the channel selection for the plotting interface using a standard table which also shows basic statistical analysis results allowing to inspect the beta-value of the oxy-hemoglobin (HbO) and deoxy-hemoglobin (Hb) time courses in individual channels based on a real-time general linear model (GLM) calculation. It is also possible to average selected channels and get a separate analysis and plot for the average. In the bottom of this interface section, one can set specific contrasts of each available condition defined in the experiment. Part three of the interface handles the preprocessing of the raw or converted HbO/Hb data. More details about the filtering options are described in the next section of this paper. In the bottom of this interface section, basic information about the experiment and settings are available.

Most of the interface (see number 4 in Fig. 1) is used to visualize and inspect time course data. There are two different types of data shown. The first one is presented in the top left (individual time course of selected channels, number 5 in Fig. 1), top right (event related averages of each condition for each selected channel, number 6 in Fig. 1), and bottom left (combined overview of all channels, number 7 in Fig. 1) of this interface section. These parts represent the individual time courses of the channels and allow to inspect each channel individually while at the same time provide an overview of all channels which makes it possible to check for overall artifacts in the data or in individual channels. The event related average plot gives more insights in condition-based response profiles allowing to compare different tasks as well as overall performance and data quality.

In the bottom right of this section, a layout overview is presented which shows the sources, detectors, and the respective channels on a predefined montage (number 8 in Fig. 1). The source and detector positions are stored with respect to the underlying montage image and can be individually adapted using an integrated layout manager or loaded from the stored information if reanalyzing a dataset (stored with NIRStar software bundled using an NIRx device).

The configuration of Secs. 1–3 is very flexible, allowing to choose where to place each part or to have it as a floating interface for multiscreen configurations.

### 2.2 Real-Time Preprocessing

In comparison to offline analysis, the major constraint of real-time analysis is to achieve a constant calculation time from time point to time point that is shorter than the sampling interval of two consecutive measurements during the whole experiment. This requirement is especially important for neurofeedback and BCI experiments using data from extended time windows or even the full-time course to calculate values for visualization and classification. The constant calculation time constraint is required for all subparts in the moment-to-moment processing pipeline including I/O operations, conversion from raw to HbO/Hb signals, preprocessing, analysis and neurofeedback/BCI calculation, and presentation, and the sum of operation times of these steps must be smaller than the sampling interval.

Turbo-Satori provides online HbO/Hb concentration value calculations from raw wavelength data using the modified Beer–Lambert law (MBLL).^18^ The parameters for concentration changes are based on the work of Essenpreis et al.^19^ who performed a study on adults to calculate the across-subject average differential path length (DPF) and the molar extinction coefficients (ϵ) from Gratzer,^20^ see Table 1.

**Table 1:** Parameters to calculate concentration changes using MBLL.

	${\mathrm{DPF}}^{\lambda }$	$\epsilon_{{\mathrm{HbO}}_{\lambda }}(\frac{l}{\mathrm{cm}\;\mathrm{mmol}})$	$\epsilon {(}_{{\mathrm{Hb}}_{\lambda }}\frac{l}{\mathrm{cm}\;\mathrm{mmol}})$
$\lambda_{1}=760\;\mathrm{nm}$	6.40	1.4865865	3.843707
$\lambda_{2}=850\;\mathrm{nm}$	5.75	2.526391	1.798643

Since the HbO/Hb concentration values need a baseline of raw data before they can be calculated in real time for future time points, the program initially shows raw data and switches to the HbO/Hb data display as soon as the baseline is available. As default, the baseline is calculated from the first 200 received values, but this can be changed using the “Hb/HbO baseline calculation period, “begin” and “end” fields in the “analysis settings” setup dialog. Raw wavelength and converted HbO/Hb data have their own preprocessing subpanels. The values of the full channel plotter and especially of the selected (and event-related) channel plotter should be scaled so that they fall within −1 and +1 (the range of the y-axis). Preprocessing options allow to detrend the raw data and apply a moving average-based low-pass filter with an order up to five. The order defines a repeated application of the low-pass filter on the data for stronger high-frequency noise removal. The HbO/Hb data can be low- and high-pass filtered in real time using a variety of different infinite impulse response (IIR) filters like the exponential moving average filter and a simple moving average filter. Implementations of RBJ Biquad, Butterworth, Chebyshev, Elliptic, Bessel, and Legendre filter designs are based on the work of Ref. 17, including low-pass, high-pass, bandpass, and bandstop transformations for most of the filters. This allows to adapt the filtering to each participant separately, giving the individual advantages and drawbacks of each filter type.^21^^,^^22^

To verify the effect of the used filtering settings, a frequency spectrogram is available showing the magnitude response for frequencies in a range from 0.0 to 1.4 Hz. The frequency plot is updated in real time and displays the frequency spectrogram of the selected channel for HbO and/or Hb. In addition to the real-time filtering procedures, a motion correction approach described in Ref. 23 is implemented which is based on the negative correlation of oxygenated and deoxygenated hemoglobin dynamics.^23^ This approach is very well suited for real-time calculation because of three major characteristics which are essential for real-time signal quality improvement. The correction at a specific point in time only relies on the data before and does not need data later than the current point, the computation time for this procedure is low and constant and it does not require user interaction because the method can be used in an automatic fashion.^23^ The method is applied after the MBLL and filtering of the data. The motion correction can be turned on using the motion correction check box in the HbO/Hb preprocessing field. For further performance details, refer to Ref. 23.

### 2.3 Real-Time Data Analysis

The analysis performed in Turbo-Satori is based on a real-time implementation of the GLM, the recursive least squares GLM (rlsGLM).^24^ Fitting an ordinary least squares (OLS) GLM is the same as finding estimates of the beta values minimizing the sum of squared error values (e). y represents the HbO or Hb data

$$e^{\prime }e=\overset{N}{\underset{t=1}{\sum }}e_{t}^{2}={\left(y-X\beta \right)}^{\prime }(y-X\beta ),$$ (1)

$$=y^{\prime }y-2\beta^{\prime }X^{\prime }y+\beta^{\prime }X^{\prime }X\beta ,$$ (2)

$$\frac{dS}{d\beta }=-2X^{\prime }y+2X^{\prime }X\beta =0.$$ (3)

The solution can be directly calculated as

$$\beta ={\left(X^{\prime }X\right)}^{-1}X^{\prime }y.$$ (4)

For the rlsGLM, the beta values (β) and inverted $X^{\prime }X$ design matrix can be updated incrementally using only information of the new time point with the following recursive equations:

$$\beta_{t+1}=\;\beta_{t}+{\left(X_{t}^{\prime }X_{t}\right)}^{-1}\frac{\left(y_{t+1}-x_{t+1}\beta_{t}\right)}{1+x_{t+1}^{\prime }{\left(X_{t}^{\prime }X_{t}\right)}^{-1}x_{t+1}},$$ (5)

$${\left(X_{t+1}^{\prime }X_{t+1}\right)}^{-1}={\left(X_{t}^{\prime }X_{t}\right)}^{-1}-\frac{{\left(X_{t}^{\prime }X_{t}\right)}^{-1}x_{t+1}x_{t+1}^{\prime }{\left(X_{t}^{\prime }X_{t}\right)}^{-1}}{1+x_{t+1}^{\prime }{\left(X_{t}^{\prime }X_{t}\right)}^{-1}x_{t+1}}.$$ (6)

Since the ${\left(X^{\prime }X\right)}^{-1}$ term is the same for all channels, it can be precomputed before solving the β for individual channels reducing the calculation time for each new data point in time.

In its standard formulation, rlsGLM results in the same beta estimates as a standard GLM over the whole time course up to the current point in time.

The GLM is calculated for each channel individually and for the average of the selected channels. The resulting beta values of the calculations are represented in the channel selection and analysis window as values as well as color coded in each channels row header. The user can change the thresholds for the color coding using the threshold spin boxes below the table. This allows a quick inspection of all channels and makes it easy for the user to select the most promising channel. A source of confounding noise in fNIRS analysis is serial correlations. Recent studies have proposed different methods to correct for this noise using offline^12^^,^^23^ and real-time^25^ data analysis. In this real-time analysis approach, the computed beta values are used for qualitative judgments, which are unaffected by serial correlations. The GLM may also be used to estimate single-trial responses (beta values) in the context of classifier applications.

The results are also shown in the connection color and strength of a source and a detector in the layout view. This view allows a spatial inspection of the activation on a predefined montage or image. In some cases, it is even useful to take a picture of the fNIRS cap on a participant’s head and directly point the sources, detectors, and channels onto this image allowing an immediate translation from activity patterns to source–detector locations on the participants head.

### 2.4 Real-Time Multivariate Pattern Classification

Multivariate pattern classification (MVPC) is gaining increasing interest in the neuroimaging community for both offline and real-time fNIRS data analysis, because it allows detecting differences between conditions with higher sensitivity than conventional univariate analysis by focusing on the analysis and comparison of distributed patterns of the activity. In such a multivariate approach, data from many sources (e.g., channels in fNIRS and EEG, voxels in fMRI) are jointly analyzed. The high sensitivity of MVPC allows “brain reading” applications that aim to decode (predict) specific mental states or representational content from activity patterns. After performing a training phase, the decoding/prediction phase requires little computational load and it is, thus, suitable for real-time BCI applications including the decoding of mental states. Turbo-Satori uses MVPC based on the widely used SVM learning algorithm. The library used in Turbo-Satori is the LIBSVM library.^26^ In the “tools” menu, the “SVM training” item can be used to open the “multichannel pattern classification” dialog allowing to train a SVM on the data from one or more completed runs of a real-time session (currently only one training run is supported); the dialog can also be used to perform offline testing, e.g., on the data of a subsequent run. After the training phase, the “real-time SVM classification” dialog can be used to start trial-by-trial online classification producing prediction values that indicate to which class a distributed activity pattern belongs according to the information extracted by the classifier.

**Fig. 2:**
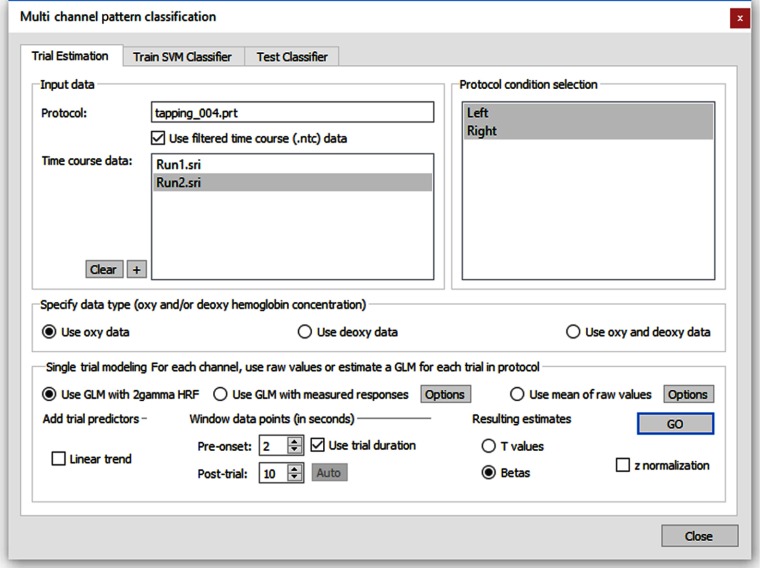
Multichannel pattern classification dialog. The trial estimation tab is selected, allowing control of the estimation of response values per trial.

The “multichannel pattern classification” dialog as shown in Fig. 2 can be used to train SVMs to associate distributed activity patterns with class labels that correspond to two or more conditions (“classes”) of a paradigm.

**Fig. 3:**
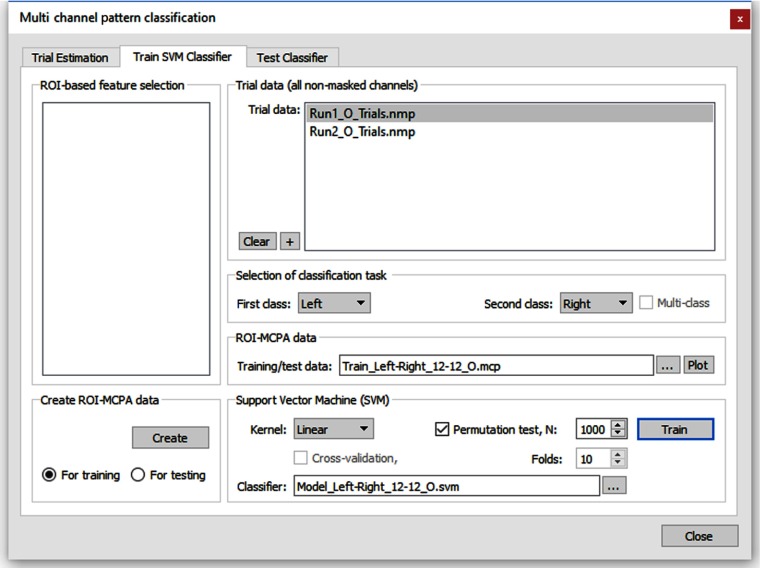
Multichannel pattern classification training dialog. Here, the train SVM classifier options are shown, enabling specification of the classification task and channels used for training.

The “trial estimation” tab (Fig. 2) is used to specify how responses should be estimated for individual trials at each channel. The “train SVM classifier” tab (see Fig. 3) is used to create training data suitable for SVMs and to perform the actual training process.

The classifier supports multiclass problems, i.e., distributed patterns may be assigned to two or more different conditions. The trained classifier can then be tested on completed run data using the “test classifier” tab. For real-time classification, the “real-time SVM classification” dialog is used. In order to obtain input patterns for different classes, response values are estimated for each individual trial using a standard OLS-GLM. Estimated single-trial responses across relevant channels (e.g., all nonmasked channels of a montage) then form the feature vectors used to train or test the classifier. An estimated trial response might be as simple as the activity level at a certain time point (e.g., at the time of the expected hemodynamic peak response) or the mean response of a few measurement points around the peak response relative to a prestimulus baseline. In Turbo-Satori, however, trial-related time points are integrated by running single-trial GLMs to estimate beta values using an expected hemodynamic response shape. The estimated single-trial responses (beta values) across channels form multivariate patterns that can be used for training runs and for online classification at the end of a trial. For a more robust fit of the model, a linear trend confound predictor may be added (next to the constant predictor and the main predictor) for estimating (and removing) a linear trend. The single-trial channel estimates may be specified as either t values, beta values, or percent signal change beta values. The beta values’ option is selected as default. As default, the time course data within each trial window is z-normalized, which can be turned off if raw time courses should be used. The “plot” button can be used to create a visualization of the created training data (see Sec. 3 for snapshot).

To test the performance of the classifier, we conducted an experiment using a finger tapping paradigm described in Sec. 2.6, including two training runs and one test run. A subset of 8 sources and 8 detectors incorporating 20 channels from the finger tapping paradigm was used in this experiment. The subset includes sources 2,3,4,6,12,13,14,16 and detectors 1,2,4,5,11,14,15,16, of the used sources and detectors described in Sec. 3.1. The channel number is based on subset source–detector pairs sorted in increasing numerical order (e.g., channel #1: source 2, detector 1, channel #2: source 2, detector 2, …). The first 10 channels (1 to 10) are placed on the left hemisphere and the 10 last channels (11 to 20) are placed on the right hemisphere. Only these 20 channels were used to train the classifier. A portable fNIRs system (NIRSport 8×8, NIRx Medizintechnik GmbH, Berlin, Germany) was used in combination with an fNIRs cap having the proper size for each individual participant. The sampling rate was 7.8125 Hz.

### 2.5 Neurofeedback Calculation

**Fig. 4:**
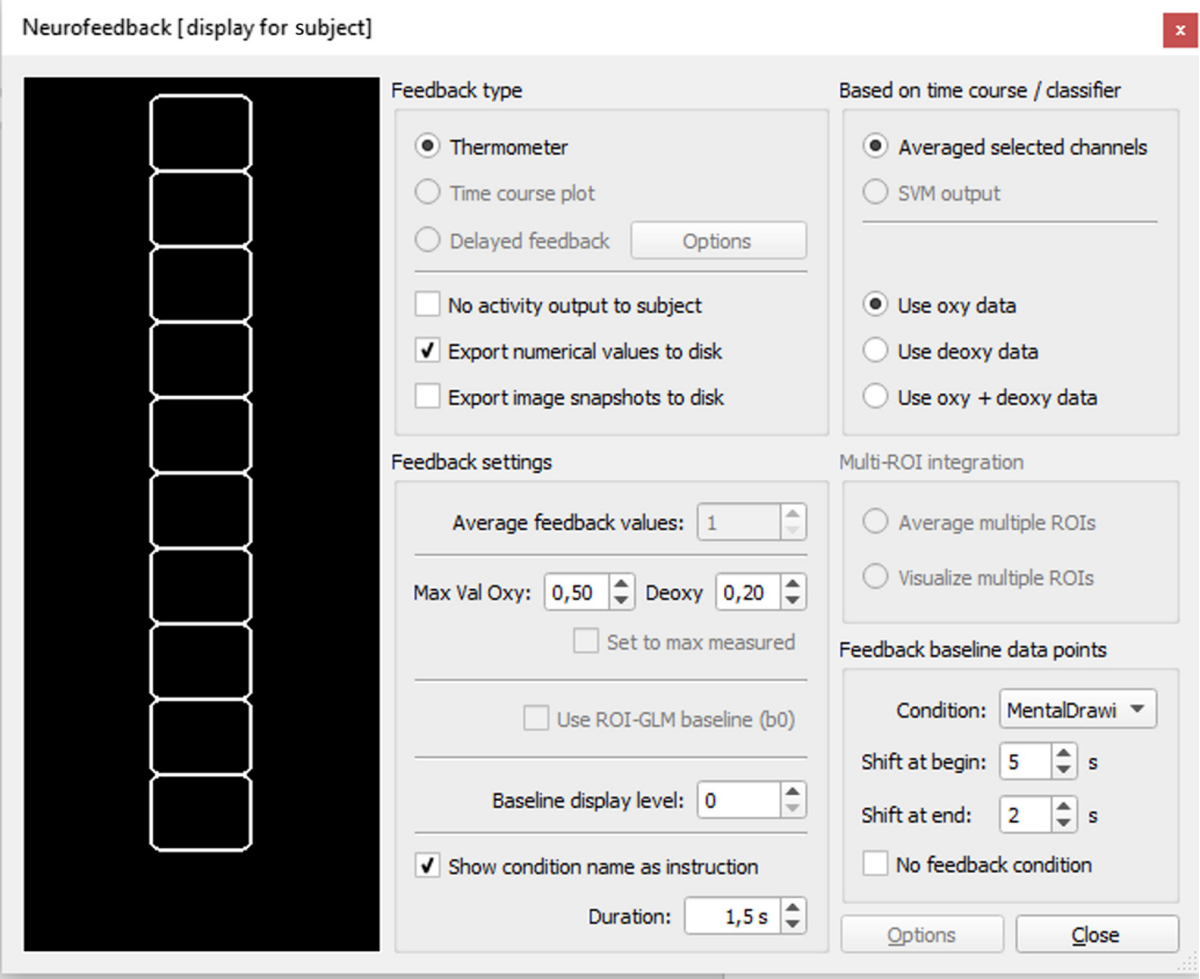
The neurofeedback dialog.

For many (clinical) neurofeedback applications, it is desirable to visualize, e.g., in a thermometer, the mean activation level from selected channels during a modulation period relative to a baseline level, and to present the activity visualization to the participant as feedback. The neurofeedback dialog, Fig. 4, can be used to select one channel or the average of multiple channels for feedback calculation and visualization and to prepare details about the feedback display.

In this section, details about the calculation of neurofeedback signals from selected channels and how calculation is controlled are described using options in the “feedback settings” field in the neurofeedback dialog. Values for neurofeedback are calculated by subtracting the last baseline (BL) from the current scaled oxy/deoxy value. Note that deoxy values are inverted so that a negative deflection results in a positive feedback signal. In detail, the calculations are as follows:

$${\mathrm{fb}}_{\mathrm{oxy}}=({\mathrm{oxy}}_{\text{value}}-{\mathrm{oxy}}_{\mathrm{BL}}),$$ (7)

$${\mathrm{fb}}_{\mathrm{deoxy}}=-1*({\mathrm{deoxy}}_{\text{value}}-{\mathrm{deoxy}}_{\mathrm{BL}}),$$ (8)

$${\mathrm{fb}}_{\text{combined}}=\frac{1}{2}*[({\mathrm{oxy}}_{\text{value}}-{\mathrm{oxy}}_{\mathrm{BL}})-({\mathrm{deoxy}}_{\text{value}}-{\mathrm{deoxy}}_{\mathrm{BL}}).$$ (9)

Note that Eqs. (7)–(9) assume a negative correlation between the patterns of HbO and Hb. This results in a percent signal value that is used to fill the thermometer display. To convert this value into a corresponding fill level of the thermometer, the resulting value is related to the “max val oxy” and “deoxy” values as specified in the max val oxy and max val deoxy field. If, for example, the calculated ${\mathrm{fb}}_{\mathrm{oxy}}$ value is 0.25 and the max val oxy value is set to 0.5, the thermometer is half filled, i.e., 5 out of 10 rectangles will be filled since $\mathrm{fb}/\max\_{\mathrm{val}}_{\mathrm{oxy}}=0.5$. To find good values for the maximum, it might be useful to observe achievable neurofeedback amplitudes for specific channels in localizer or test runs.

Because of the sluggishness of the fNIRS (BOLD) response, the values defined by a baseline interval should not be directly used as defined in the protocol, but should take the hemodynamic delay into account. In order to protect the BOLD decay from a previous modulation condition, values at the beginning of the baseline condition need to be excluded. The number of points to excluded can be specified with the “shift at begin” spin box. If the previous baseline condition occurred, for example, from time points (s) 50 to 70 (20 s), the actual values considered for baseline calculation would be from 55 to 70 in case of value 5 for the shift at the beginning of the baseline interval. In a similar way, one can also extend points “to the right side” at the end of a baseline interval since it takes some time until the BOLD signal rises in the modulation block. Since it is expected that the signal already starts to rise after about 2 to 3 s, the shift into the modulation interval should be, however, smaller than at the beginning of the baseline condition. Using 1 s for the shift at end parameter results in the final interval 55 to 71 for the baseline period. The signal values of the identified data points within this period (16 s in the example) will be averaged to obtain the baseline level BL for the subsequent modulation block. Note that 4 s are minimally required for a premodulation baseline (at least 10 are recommended); otherwise, no feedback output is produced in the thermometer display.

### 2.6 Real-Time Preprocessing and Analysis Verification

We used data from four experiments to verify the calculation times for the whole preprocessing and analysis using second-order real-time Butterworth low- and high-pass filters and rlsGLM calculation with two main predictors. The four experiments were analyzed *post hoc* using the same routines as used during real-time measurements allowing for a direct translation. The first experiment (Fig. 8, yellow, 20 channels [10.4167 Hz]) consisted of 224,990 data points which were generated automatically simulating an ∼6-h experiment. The second experiment (Fig. 8, blue, 64 channels [7.8125 Hz]) was a mental drawing paradigm which consisted of 3291 data points and took ∼7  min. The third and fourth experiments were data from the NIRx support website^27^ including both a left- or right-hand finger tapping experiment. The first of these two experiments (Fig. 8, orange, 256 channels [7.8125 Hz]) consisted of 2750 data points and took ∼6  min and the second experiment (Fig. 8, gray, 256 channels [3.91 Hz]) consisted of 2460 data points which required ∼10  min. These two datasets were also used to show the performance of the rlsGLM and user interface as describe in the results section.

### 2.7 Experimental Setup

**Fig. 5:**
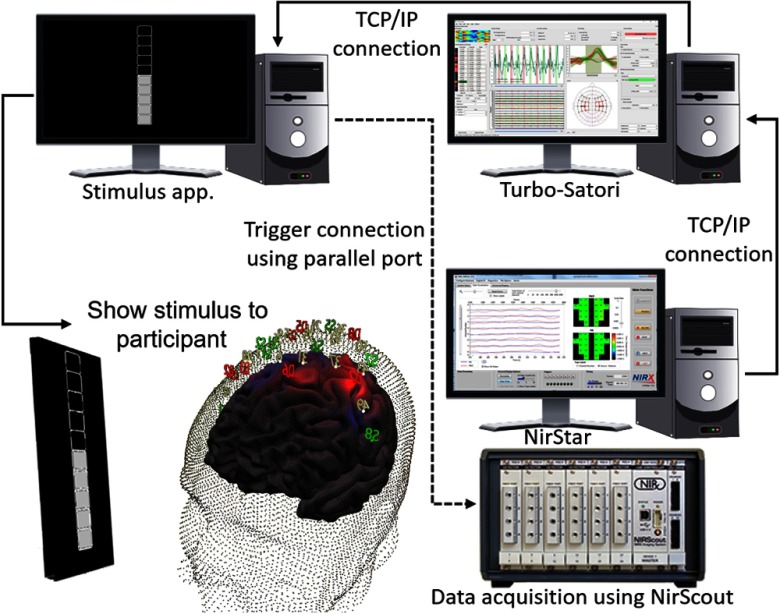
Setup of real-time fNIRS neurofeedback experiment using Turbo-Satori.

BCI and especially neurofeedback experiments can require a complex setup involving multiple devices and software. It is, therefore, important to clarify the connections from and to Turbo-Satori as well as the position in an example setup as shown in Fig. 5.

The data acquisition is based on an NIRx fNIRS system (NIRScout, NIRSCOUTX, or NIRSport) which is operated by a computer or laptop running NIRStar (version 14.2)27. The NIRx device is connected via a universal serial bus cable to the computer with NIRStar. Turbo-Satori is installed on a separate computer which connects to the computer running NIRStar via a TCP/IP Ethernet connection. The two software packages use the NIRx network software development kit to communicate in real-time and exchange data between NIRStar and Turbo-Satori. For BCI or specifically neurofeedback applications, the stimulus presentation software needs to read or receive the BCI or neurofeedback information in real time. In this example setup, the stimulus presentation software also runs on a separate computer. This computer is also connected to the computer running Turbo-Satori using a TCP/IP-based Ethernet network connection. The data are exchanged using a self-designed network interface enabling real-time access to raw and preprocessed fNIRS data as well as protocol and statistical information. This interface is available for software tools like Expyriment,^28^ BrainStim,^29^ or MATLAB (MATLAB, The MathWorks Inc., Natick, Massachusetts). The prepared stimuli and BCI or neurofeedback information are then presented to the participant.

The separation of all three software tools ensures a fast processing of each element in the chain and ensures compliance to the time critical requirements of these kinds of experiments.

## 3 Results

### 3.1 Finger Tapping Example

**Fig. 6:**
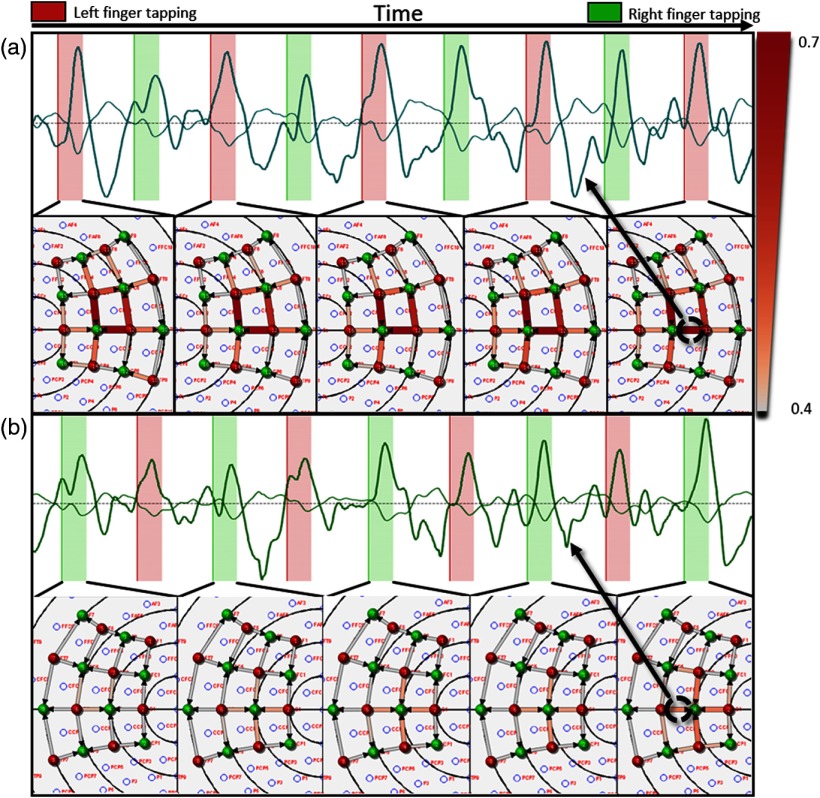
Incremental results of the left and right finger tapping experiment. (a) The right hemisphere pattern and the oxy (bold line)/deoxy (thinner line) time course of the channel covering C4. (b) The left hemisphere pattern and oxy (bold line)/deoxy (thinner line) time course of the channel covering C3. The strength of the edges between sources (red points) and detectors (green points) represent the beta values using the HbO time courses for the GLM calculation in this example.

The rlsGLM analysis revealed different patterns for left and right finger tapping visualized in the layout view of Turbo-Satori. The task specific patterns on the right hemisphere for left finger (red task period) tapping and on the left hemisphere for right finger tapping (green task period) appear after three task epochs on the right hemisphere during the left tapping task and after four task epochs on the left hemisphere for the right tapping task using a fixed beta threshold. The minimum beta threshold was set to 0.40 (white color and thin lines) and the maximum color coding (red) and strength was set to 0.70. For both tasks, a separate task versus rest contrast was selected. After each task block, a state of the current layout was extracted and put together in Fig. 6.

After a few repetitions of each task, one can see a pattern evolving representing the channels exceeding the selected beta threshold. Also, a difference in the pattern strength is visible; in Fig. 6(a) the right hemisphere for left finger tapping and in Fig. 6(b) the left hemisphere for right finger tapping exhibit a larger response strength. The beta values of the offline analysis were highly correlated compared to the online analysis having an average Pearson correlation coefficient of 0.999 with significance at P<0.0001. Comparing the neurofeedback levels and the underlying preprocessed fNIRS signal (for HbO, Hb, and mean) resulted in an average Pearson correlation of 0.9311 with significance at P<0.0001.

### 3.2 Support Vector Machine Analysis of Finger Tapping Experiment

**Fig. 7:**
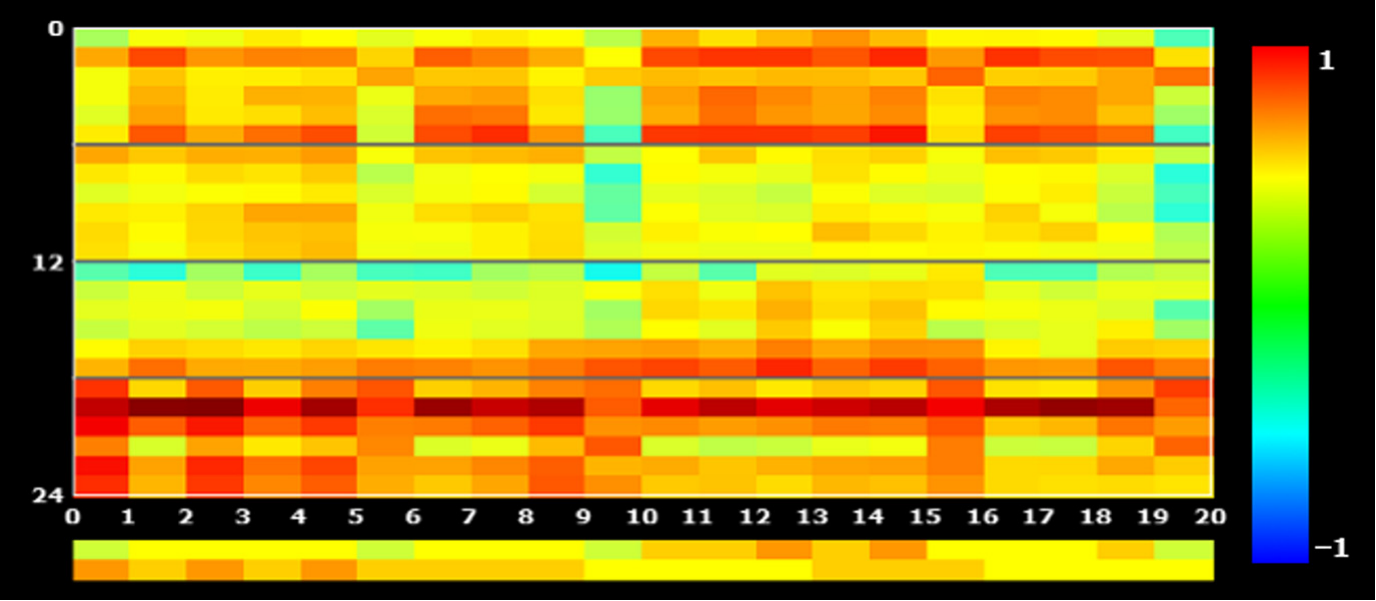
Visualization of the finger tapping training dataset in the training pattern plot. Rows in the upper part represent patterns of single trial with black lines separating different classes and runs (first run class 1, first run class 2, second run class 1, second run class 2). The colored rectangles indicate the value of the estimated responses within a pattern for a given channel (negative values green-to-blue, positive values yellow-to-red). The rows at the bottom of the graph show the mean values averaged over the trials of the two available classes (left and right tapping).

In Fig. 7, the trained classifier is shown. The y-axis represents the training patterns with black lines separating the different classes for each run (first run class 1, first run class 2, second run class 1, second run class 2); the x-axis represents the channels used for training (20 channels in this example). The colored rectangles indicate the value of the estimated responses within a pattern for a given channel (negative values green-to-blue, positive values yellow-to-red). The rows at the bottom of the graph show the mean values averaged over the trials of the available classes. In this example, one can clearly see in the average plot a pattern in the trial responses. For the first class (left finger tapping), the last 10 channels (11 to 20, placed on top of the right hemisphere) show a stronger response and the first 10 channels (1 to 10, placed on top of the left hemisphere) show a larger response for the second class (right finger tapping).

We employed a leave-one-run-out splitting procedure to cross-validate the classification performance between left and right finger tappings using a 1000 folds’ permutation test. For this dataset, using two training runs (12 trials per condition) and one test run, an accuracy of 100% was reached having a significant accuracy (p<0.01). The 95th percentile of each 1000 permutations test was 69.36%.

### 3.3 Calculation Times

**Fig. 8:**
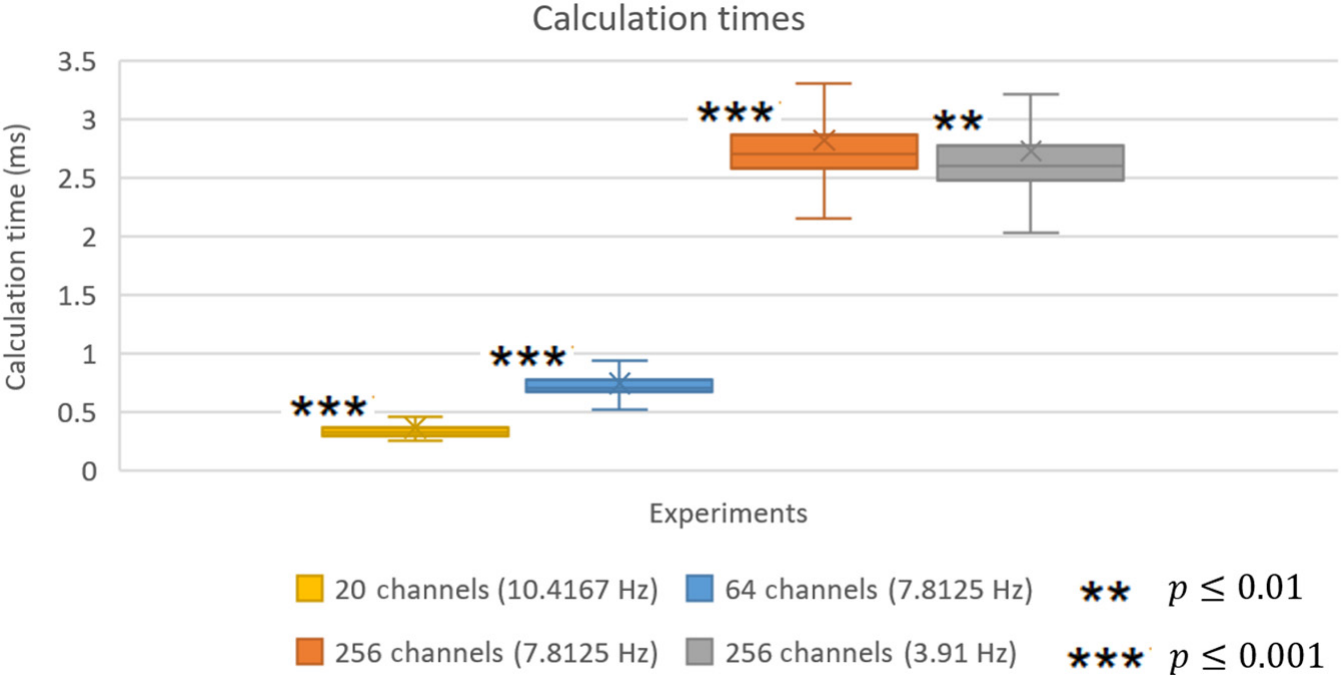
Calculation times for the four conducted experiments with varying number of channels and sampling rate. Asterisk indicates no trend in calculation times.

All experiments were analyzed successfully in Turbo-Satori using a standard laptop computer with 3.0 GHz Intel^®^ Core™ i7-5500U CPU, 8 GB RAM and 512 MB SSD storage. The calculation times were much lower than the respective sampling rates. For the first experiment, an average calculation time of 0.361 ms (min 0.246 ms, max 2.249 ms, std. 0.0841) was achieved using a sampling rate of 10.41467 Hz (∼96-ms interval). The second experiment was performed with a mean calculation time of 0.741 ms (min 0.443 ms, max 4.480 ms, std. 0.164) with a sampling frequency of 7.8125 Hz (128-ms interval). For the third experiment, a calculation time of 2.661 ms (min 1.496 ms, max 15.544 ms, std. 0.509) was achieved using a sampling rate of 7.8125 Hz (128-ms interval). The last experiment performed similarly having an average calculation time of 2.753 ms (min 1.495 ms, max 16.712 ms, std. 0.503) using a sampling rate of 3.91 Hz (∼255-ms interval). All experiments did not show any significant linear increase in calculation time over the whole dataset (see asterisks in Fig. 8). An overview of these results is shown in Fig. 8.

## 4 Discussion

BCIs and especially neurofeedback applications using fNIRS have gained increasing importance in recent years. Therefore, software is needed that helps researchers and clinicians to easily use state-of-the-art analysis methods and to apply these techniques in broad research and increasingly in clinical fields. Turbo-Satori has been developed to fulfill these needs by integrating a fast calculation engine implemented in C++ with an easy to use interface that provides useful information about channel-level signal quality. The software offers analysis methods including statistical and classification (SVM) tools that are useful for BCI applications. Furthermore, calculation and visualization tools are provided that enable neurofeedback applications.

The implemented incremental GLM, which has been used in real-time fMRI applications for many years, could potentially be used as a verification of a participant’s performance and speed up the experiments by giving more insights during the experiment. This would allow, for example, to stop a localizer experiment earlier in the case where a required performance level is not reached, saving time that can be used to better instruct the participant. A standard (windowed) GLM is used to estimate single-trial channel responses as part of the classification tools. The (incremental) GLM can also be used to regress out confounds (e.g., drifts) from the data providing cleaned input data for neurofeedback and classification subroutines. The high sampling frequencies used in fNIRS experiments introduce a significant temporal autocorrelation within the time course, which can lead to inaccurate estimation of the signal degrees of freedom and error covariance using OLS-based algorithms.^30^ Therefore, the current version of Turbo-Satori reports beta-values as a default output. Nevertheless, there are popular methods to mitigate this effect including data “precoloring”^31^ and “prewhitening.”^30^ In a recent paper, Barker et al.^25^ presented an online least square estimation algorithm incorporating the “prewhitening” technique for real-time fNIRS. These tools are planned to be integrated in a future version of Turbo-Satori.

While Turbo-Satori already offers many relevant features, further improvements and integration of new tools for real-time fNIRS processing and analysis are needed. To allow adding custom calculations, a plugin interface has been created enabling software developers to extend the capabilities of the software.

The broad spectrum of different kinds of IIR filters offer a high flexibility for the researcher to easily select a good filter and corresponding settings during data acquisition for each individual participant. As shown in Ref. 12, these individual filterings can be a crucial factor if the data quality is not optimal and is very noisy.

The sampling rate of fNIRS data acquisition can be selected in the hardware setup of the NIRx NIRs device. It allows to freely decide based on the number of sources, detectors, and steps, how fast the data should be acquired. For real-time fNIRS, the sampling frequency is in principle the maximum time that is available to still perform the analysis and preprocessing in real time. The calculation time strongly depends on the number of channels used in the analysis. Using only 20 channels resulted in a much faster calculation allowing for an overall higher sampling rate in the future. Using only the channels of interest in the data analysis would, therefore, be the best option for real-time applications, whereas for the localizer online analysis all channels could be considered, ensuring the best selection for the BCI or neurofeedback experiment.

Also in the field of neurofeedback-based treatments, fNIRS has gained more attention and many studies have recently been published.^9^^,^^32^^,^^33^ This shows the importance and need of more tools for real-time processing which will allow more researcher to focus their research on real-time fNIRS applications and treatments as was the case for real-time fMRI studies.^34^ The standardization of the accessibility of fNIRS data is also very important to facilitate the applications of real-time fNIRS experiments using different hardware to record the fNIRS signal. In this paper, we based our developments on hardware from NIRx which uses a proprietary interface to gain access to the fNIRS data in real time. An alternative would be the use of an already elaborated library like the lab streaming layer^35^ for the unified collection of measurement time series in research experiments. One can gain access to the software in a collaborative environment since new tools and features are continuously added. It is also possible that the software may become commercially available in the future.

## 5 Conclusion

We presented Turbo-Satori, a feature rich software toolbox for real-time fNIRS neurofeedback and BCI applications. An example setup was proposed using a clear differentiation between data acquisition, processing, and presentation ensuring a good workflow and straightforward interaction with the different tools. With data from four experiments, we showed the stability and reliability of the calculation performance from preprocessing and analysis methods ensuring a durable performance even for very long experiments running for several hours.
